# Drivers and barriers of vaccine acceptance among pregnant women in Kenya

**DOI:** 10.1080/21645515.2020.1723364

**Published:** 2020-03-25

**Authors:** Nancy A. Otieno, Fredrick Otiato, Bryan Nyawanda, Maxwel Adero, Winnie N. Wairimu, Dominic Ouma, Raphael Atito, Andrew Wilson, Ines Gonzalez-Casanova, Fauzia A. Malik, Marc-Alain Widdowson, Saad B. Omer, Sandra S. Chaves, Jennifer R. Verani

**Affiliations:** a Center for Global Health Research, Kenya Medical Research Institute, Kisumu, Kenya; b Rollins School of Public Health, Hubert Department of Global Health, Emory University, Atlanta, GA, USA; c Division of Global Health Protection, Center for Global Health, Centers for Disease Control and Prevention, Nairobi, Kenya; d Institute of Tropical Medicine, Antwerp; e Yale Institute for Global Health; f Department of Internal Medicine (Infectious Diseases), Yale School of Medicine; g Department of Epidemiology of Microbial Diseases, Yale School of Public Health; h Influenza Program, National Center for Immunization and Respiratory Diseases, Centers for Disease Control and Prevention, Nairobi, Kenya

**Keywords:** pregnant women, drivers, barriers, vaccine acceptance, uptake

## Abstract

Maternal vaccination coverage remains suboptimal globally and is lowest in low- and middle-income countries. Attitudes toward maternal vaccines have been characterized in middle-high income settings, however data from African countries are limited. We assessed drivers and barriers of vaccine acceptance among pregnant women in Kenya. We conducted a cross-sectional survey among pregnant women aged 15–49 y. We enrolled a convenience sample of women presenting for antenatal care at seven health-care facilities in four diverse counties (Nairobi, Mombasa, Marsabit, Siaya) of Kenya and from the community in two counties (Nairobi, Siaya). We described frequencies of socio-demographic characteristics of participants and their knowledge, attitudes, and beliefs regarding maternal vaccination. We enrolled 604 pregnant women with a median age of 26.5 y, of whom 48.2% had primary education or less. More than 95% agreed that maternal vaccines are “important for my health” and that getting vaccinated is “a good way to protect myself from disease”. The most commonly cited reason in favor of maternal vaccination was disease prevention (53.2%). Fear of side effects to mother/baby (15.1%) was the most frequently reported potential barrier. Influenza vaccine is not in routine use in Kenya; however, 77.8% reported willingness to accept influenza vaccination during pregnancy. Maternal vaccination is well accepted among Kenyan pregnant women. We identified the provision of adequate vaccine information and addressing safety concerns as opportunities to improve maternal vaccine uptake. The expressed willingness to receive a vaccine not currently in routine use bodes well for implementation of new maternal vaccines in Kenya.

## Introduction

Maternal vaccination is an important strategy to prevent maternal, neonatal, and infant disease.^1-3^ Despite evidence on the safety and effectiveness of maternal vaccines, challenges to achieving high vaccination coverage during pregnancy still exist globally.^4^ The World Organization (WHO) recommends tetanus toxoid (TT) vaccination for all pregnant women until they receive required doses to achieve full protection; however, globally, coverage for two or more TT-containing vaccines among pregnant women is 73%,^5^ while the universal target requires ≥80% coverage that is needed to achieve and maintain maternal and neonatal tetanus elimination.^6^ The proportion of pregnancies adequately protected against tetanus is lowest in Africa (60%).^7,8^ In Kenya, TT is the only maternal vaccine included as part of the Kenya Expanded Programme on Immunization, yet the uptake of two or more TT doses among pregnant women in the last Kenya Demographic and Health Survey (KDHS, 2014) was 51.1%,^9^ which is far below the universal target for TT coverage. Although Kenya achieved the elimination of maternal and neonatal tetanus in 2019,^10^ this low coverage of maternal TT vaccination presents a substantial risk to the health and well-being of pregnant women and young infants.

Acceptance of vaccines and vaccine hesitancy is influenced by a wide range of factors that may vary by time, place, and type of vaccine.^11,12^ Acceptance of vaccines among pregnant women presents additional and more complex issues since both mother and baby are affected.^13^ Lower levels of education, lack of knowledge on vaccination during pregnancy and other socioeconomic factors like high parity and low income have been identified as barriers to maternal vaccination in low- and middle-income countries (LMICs); however data on maternal vaccine acceptance from African settings are very limited.^4,14-16^ TT is the most widely implemented vaccine among pregnant women in LMIC.^14^ In 2012, however, the WHO recommended that countries considering initiation or expansion of seasonal influenza vaccination give the highest priority to pregnant women,^17^ and in 2015 noted that maternal vaccination against pertussis is likely to be the most cost-effective way to prevent the disease in infants too young for vaccination.^18^ Moreover, new vaccines for use in pregnant women, for example, against Group B *Streptococcus* and respiratory syncytial virus, are under development and have great potential to reduce disease burden in resource-poor settings.^19-23^ Thus, a better understanding of factors that may affect the acceptability of currently available and newly introduced maternal vaccines in LMIC, and particularly in Africa, is needed. The purpose of this study was to examine knowledge, drivers, and barriers of maternal vaccine acceptance in Kenya.

## Materials and methods

**Figure 1.**
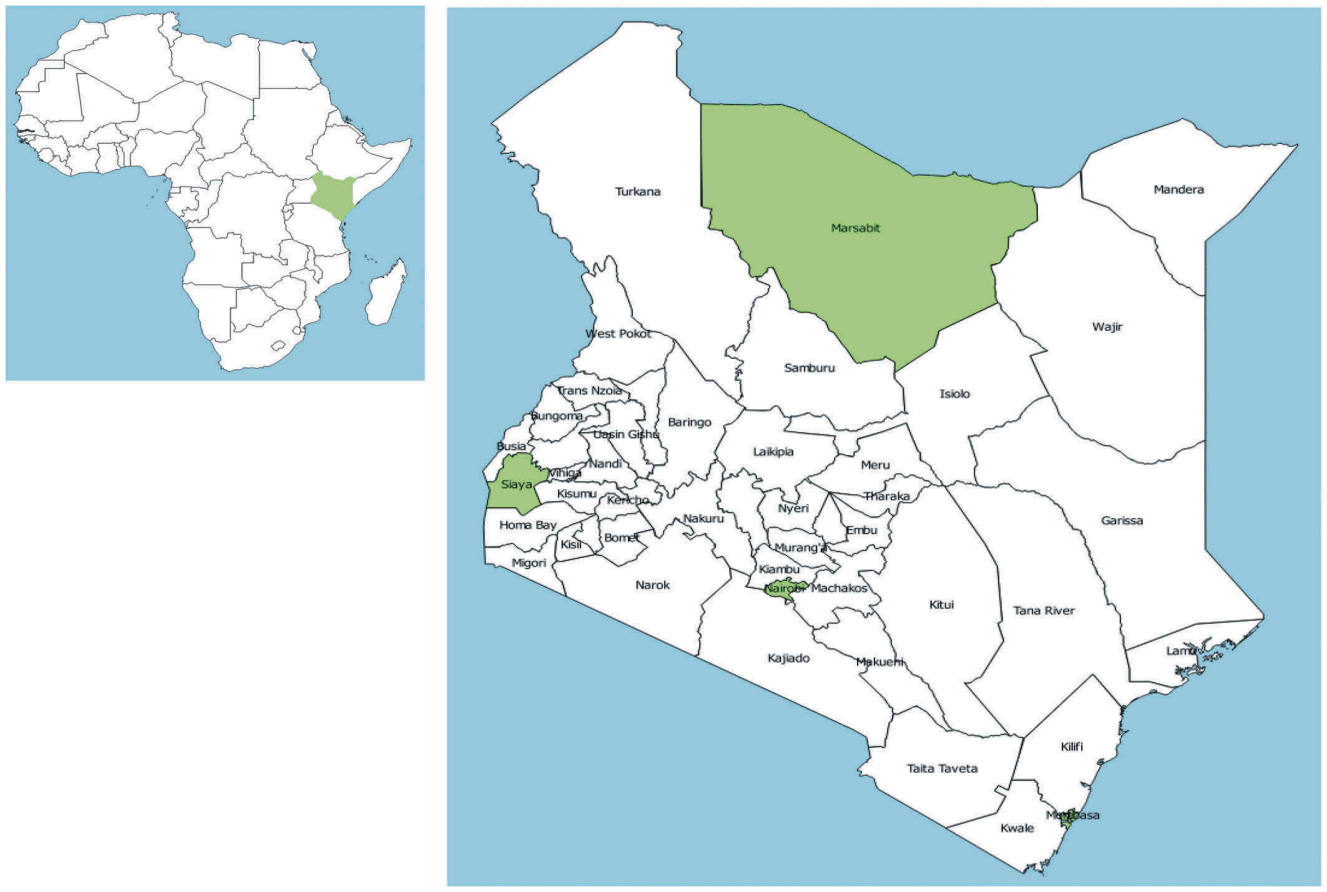
Map of Africa (left) Showing the Position of Kenya, and Map of Kenya with the Location of Counties Where the Study Took Place

This analysis was part of a larger study examining factors that shape the acceptance of maternal vaccines in Kenya. We conducted a cross-sectional survey of knowledge, attitudes, and beliefs regarding maternal vaccination among pregnant women. The survey was implemented in four counties in Kenya: Marsabit, Nairobi, Siaya, and Mombasa. Map showing the position of the counties of study implementation is shown in Figure 1.

Study site choice was guided by the geographic spread, cultural and religious diversity, and aiming for a mix of urban, sub/peri-urban, and rural settlements in Kenya. The study settings have relatively poor vaccination coverage and high burden of maternal and infant mortality.^24^ A summary of key characteristics of the study sites is shown in Table 1.

**Table 1.** Characteristics of the study sites

County	Percentage receiving antenatal care from skilled provider^a, e^	Percentage receiving two or more TT injections during last pregnancy^b, e^	Percentage whose last birth was protected against neonatal tetanus^b, c, e^	Maternal mortality (deaths per 100,000 live births^e^	Infant mortality^b, e^ (deaths per 1000 live births)	Rural/Urban	Facility where women were enrolled	Number of Pregnant women enrolled
Nairobi	97.6	60.3	83	212^b^	55	Urban	Mbagathi District Hospital	110
							Tabitha Clinic Kibera	114
							Kibera Community (referred)	46
Mombasa	99.2	64.5	83.7	328^b^	44	Urban	Coast Provincial General Hospital	86
							Tudor Health Center	18
Marsabit	75.6	44.1^d^	69.5^d^	1,127^f^	37	Peri-urban	Marsabit District Hospital	70
Siaya	97.8	44.5	70.1	692^f^	50	Rural	Siaya County Referral hospital	101
							Lwak Mission Hospital	8
							Siaya Community (referred)	51
National overall	95.5	51.1	75.6	362	39			

^a^Skilled provider includes doctor, nurse, or midwife.

^b^Regional rates used, county rates not available

^c^Includes mothers with two injections during the pregnancy of her last birth, or two or more injections (the last within 3 y of the last live birth), or three or more injections (the last within 5 y of the last birth), or four or more injections (the last within 10 y of the last live birth), or five or more injections at any time prior to the last birth.

^d^Marsabit is in the eastern region though located at and with indicators similar to northern Kenya. Regional rates presented are averages of the two regions.

^e^Kenya Demographic and Health Survey 2014; National Council for Population and Development and United Nations Population Fund

^f^Kenya Population Situation Analysis, 2013 TT = Tetanus toxoid

Women were eligible for study participation if they were pregnant, aged 15–49 y old, were residents of the study counties, and able to provide informed consent. Potential participants were recruited from antenatal clinics at health facilities in all selected counties, and from the community in two counties (Nairobi and Siaya). We aimed to enroll 600 pregnant women across all sites, including 500 from health facilities and 100 from the community. The sample size was computed under the assumption of identifying the most conservative response proportion, 50%, for a given survey variable, using PASS v11 (NCSS, LLC; Kaysville UT), using the confidence interval for one proportion module. To estimate a response proportion of 50% with a 95% confidence interval of ± 5% (i.e., 95% confidence interval 45% to 55%) with 80% power, we would need a total of 402 completed surveys; we increased the target enrollment to 600 in order to conduct certain stratified analyses (for example, among women with a prior pregnancy) with the desired precision.

For facility-based recruitment, study staff enrolled a convenience sample of pregnant women presenting for antenatal care at the main public referral hospitals in each of the counties, as well as two private, nonprofit facilities: Tabitha Clinic (located in Kibera, an informal urban settlement in Nairobi) and St. Elizabeth Lwak Mission Hospital (located in rural Siaya County). For recruitment from the community in Nairobi and Siaya, study staff identified pregnant women registered for antenatal care at a participating facility who did not present for care during the recruitment period; these women were contacted by phone and invited to participate in the study. In addition, participants were encouraged to refer other pregnant women from their communities; interested pregnant women who contacted study staff and met enrollment criteria were invited to participate. Women recruited from the community were given the option to be interviewed at home or at a nearby health facility.

We pilot tested the study questionnaire at two facilities in Nairobi county prior to study implementation. Adjustments were made on the questionnaire based on feedback from the pilot test to ensure reliability. After obtaining written informed consent, the standardized questionnaire was administered. The questionnaire covered participant demographics and obstetric history, prior experience with vaccination (for themselves and their children), sources of vaccine information and recommendations during pregnancy, beliefs about vaccine protection and perceived benefits during pregnancy, and reasons for and against vaccination during pregnancy. Although Kenya does not currently have a maternal influenza vaccination program, the questionnaire also included questions about awareness of influenza disease (including perceived risk), attitudes/beliefs on influenza vaccination and willingness to receive influenza vaccine. The questionnaire was constructed primarily through measurement on three- and five-point Likert scales, responses ranged from agree to disagree and not relevant at all to very relevant or strongly disagree to strongly agree, respectively. The survey instrument was based upon previously used questionnaires shown to have high validity^25-28^ and the compendium of survey questions developed by the WHO Strategic Advisory Group of Experts working group on vaccine hesitancy.^29^

The survey questionnaire was translated into local languages (Swahili, Luo, Gikuyu, and Borana) and back-translated into English to ensure accuracy of translations before administration. Interviews were conducted in private study offices or in households of study participants enrolled in the community. Data were collected electronically using tablets and transmitted real time onto KEMRI servers for management and storage.

Analysis was done using STATA version 13.0 (Stata Corp., College Station, TX). We described the frequencies of socio-demographic characteristics and responses to survey questions.

Ethical clearance for the study was obtained from KEMRI (SSC. 3292) and Emory University (IRB00089673) institutional review boards (IRBs), with CDC reliance on non-CDC IRB (CDC Protocol #6974.0). Informed consent was obtained from all participants before enrollment.

## Results

From October 2017 to January 2018 we enrolled 604 pregnant women, including 507 from health facilities and 97 from the community (Table 1). The median age of participants was 26.5 y, and 65 (10.8%), 290 (48.0%), and 246 (40.7%) were in first, second, and third trimester of pregnancy, respectively (Table 2). The highest education level attained was primary school for 229 (37.9%), and secondary school for 190 (31.5%). Participants were most commonly married (n = 502, 83.1%); 216 (35.8%) were housewives and 60 (9.9%) had formal employment. The predominant ethnic group was Luo (n = 295, 48.8%). Overall, 441 (73.0%) had one or more prior pregnancies; 112 (25.4%) of those had experienced a miscarriage during a prior pregnancy.

**Table 2.** Socio-demographic and pregnancy characteristics of women enrolled in the study, October 2017 – January 2018, N = 604

Characteristic	n	%
*Maternal age*		
15–24 y	224	37.1
25–34 y	333	55.1
35–49 y	47	7.8
Age (yrs), mean (sd)	26.6	(5.3)
Age (yrs), median (IQR)	26.5	(23,30)
*Gestational age*^a^		
First trimester	65	10.8
Second trimester	290	48.3
Third trimester	246	40.9
*Level of education*		
No education	62	10.3
Primary only	229	37.9
Secondary	190	31.5
College	123	20.4
*Marital status*		
Single	94	15.6
Married	502	83.1
Divorced/Separated	5	0.8
Widow	2	0.3
Don’t want to answer	1	0.2
*Primary source of income*		
Housewife	216	35.8
Small business (no premise eg. sell maize)	105	17.4
Not working^b^	82	13.6
Business owner (has premise eg. small shop)	74	12.3
Salaried worker (eg. teacher, nurse, office)	60	9.9
Skilled labor (carpenter, tailor, artisan)	38	6.3
Unskilled labor (farming, construction)	29	4.8
*Religion*		
Protestant	278	46.0
Catholic	157	26.0
Muslims	85	14.1
Traditional African Churches/traditional religion	84	13.9
*Ethnicity*		
Luo	295	48.8
Borana/Rendile/Burji/Somali	67	11.1
Kikuyu	54	8.9
Luhya	52	8.6
Swahilli/Mijikenda	37	6.1
Kamba	35	5.8
Other	64	10.6
*No. of children living in the household*, median (IQR)	2 (1,3)	
No. of children < 5 y, median (IQR)	1 (1,1)	
*No. of pregnancies including the current one*		
1	163	27.0
2	183	30.3
3	139	23.0
4	64	10.6
≥5	55	9.1
Past miscarriages (mothers on second or more pregnancies), n = 441	112	25.4
Yes		
Hospitalization during current pregnancy	32	5.3

^a^Three women did not know the gestational age of their pregnancies, n = 601

^b^Includes 44 mothers who reported being students and 13 who reported subsistence farming.

Among all participants, 361 (59.8%) had received a recommendation for vaccination during the current pregnancy, and health-care providers (n = 253, 70.1%) were the most frequent source of vaccine recommendation (Table 3). Other common sources of recommendations for vaccination during pregnancy included relatives (n = 114, 31.6%), friends/neighbors (n = 109, 30.2%), husbands (n = 102, 28.3%), and community health workers (n = 82, 22.7%). The vaccine most commonly recommended was TT (n = 277, 76.7%). The rest of the women did not know (n = 47, 13.0%), could not remember (n = 34, 9.4%) the specific vaccine recommended, or mentioned other products (n = 3, 0.8%) such as iron boosters that were not TT. Overall, 429 (71.0%) reported having received a vaccine in the current pregnancy, and among 433 women with one or more prior pregnancies, 401 (92.6%) reported having previously received a vaccine while pregnant. TT was the most common vaccine received during the current (n = 415, 96.7%) and prior (n = 347, 86.5%) pregnancies. Among those who received TT during the current pregnancy, 82 (19.8%) reported some type of complication; pain (n = 39, 47.6%) was most common. When asked about the number of vaccines they would be willing to receive during pregnancy, 101 (16.7%) participants replied no more than one, 71 (11.9%) no more than two, and 216 (35.8%) reported that they would have no limit and 140 (23.2%) were unsure.

**Table 3.** Vaccine recommendations and uptake among pregnant women enrolled in the study, October 2017 – January 2018, N = 604

Characteristic	n	%
Received recommendation to get vaccinated during current pregnancy	361	59.8
Source of vaccine recommendation^a^		
Doctor, Nurse, or other health-care providers	253	70.1
Relative	114	31.6
Friend/Neighbor	109	30.2
Husband	102	28.3
Community Health Worker	83	23.0
Through Radio, TV Or Internet/Social Media	32	8.9
Ministry of Health	14	3.9
Father of child	8	2.2
Religious leaders	6	1.7
Local leaders	4	1.1
Chemist/pharmacist	1	0.3
Others	19	5.3
Vaccine recommended during current pregnancy		
Tetanus vaccine	277	76.7
Do not know vaccine	47	13.0
Cannot remember vaccine name	34	9.4
Other^b^	3	0.8
Received vaccine during current pregnancy	429	71.03
Specific vaccine(s) received		
Tetanus vaccine^c^	415	96.7
Unknown	14	3.3
Week of pregnancy received tetanus vaccine (median, IQR)	20	(16,24)
Complications from tetanus vaccination	82	19.8
Type of complication^d^		
Pain	39	47.6
Numbness	16	19.5
Swelling	13	15.9
Bleeding	2	2.4
Fever	1	1.2
Other	12	14.6
No complications	333	80.2
Received vaccine during prior pregnancy, n = 433^e^	401	92.6
Specific vaccine(s) received		
Tetanus vaccine	347	86.5
Do not know/remember	54	13.5
Maximum number of vaccines willing to receive during pregnancy		
1	101	16.7
2	72	11.9
3	46	7.6
4	12	2.0
≥5	17	2.8
No maximum	216	35.8
Not sure	140	23.2
Stage of pregnancy woman likely to take up a vaccine		
All throughout pregnancy	108	17.9
During the first 3 months	222	36.8
During the first 6 months	211	34.9
Only during the last 3 months	63	10.4

^a^Women could reply to more than one option so total >361.

^b^Respondents reported Anti D, Iron boosters, and malaria vaccine; malaria vaccine is not given in pregnancy while Anti D and iron boosters are not vaccines.

^c^One mother reported receiving influenza, pertussis, and HPV vaccine in addition to tetanus vaccine; influenza, pertussis, and HPV vaccines are not routinely given to pregnant women in Kenya.

^d^Women could reply to more than one option for the type of complication, so total >82

^e^Denominator excludes mothers in their first pregnancy (n = 163) and those who were not sure of receiving vaccine in the past pregnancy (n = 8).

When asked about the top three reasons why a woman would receive a vaccine during pregnancy, disease prevention was the most frequent response (first priority for 53.2%, second priority for 26.5%, and third priority for 9.3%), followed by the benefit to the baby in the womb (first priority for 18.9%, second priority for 33.9%, and third priority for 25.8%) and to follow health-care provider recommendations (first priority for 17.2%, second priority for 15.4%, and third priority for 20.5%) (Table 4). When asked about the top three reasons why a woman would refuse a vaccine during pregnancy, about half answered that they would never refuse vaccination against tetanus during pregnancy (first priority for 51.0%, and second priority for 0.5%). The most frequent reason for refusal when given was if the vaccine caused side effects to a mother or baby (first priority for 15.1%, second priority for 6.1%, and third priority for 1.5%). When asked about whose benefit should be prioritized in deciding whether a pregnant woman should be vaccinated, the baby in the womb was the most common first priority (first priority for 58.8%, second priority for 33.6%, and third priority for 7.6%), followed by the mother as the most common second priority (first priority for 34.1%, second priority for 41.9%, and third priority for 24.0%).

**Table 4.** Top reasons for and against vaccination during pregnancy reported by pregnant women, N = 604

	First priority	Second priority	Third priority
Reason	n (%) n = 604	n (%) n = 604	n (%) n = 604
Reasons why a woman would receive a vaccine during pregnancy			
Vaccines help prevent diseases	321 (53.2)	160 (26.5)	56 (9.3)
The vaccine is beneficial to the baby in the womb	114 (18.9)	205 (33.9)	156 (25.8)
If a doctor, nurse or other health-care provider suggested it	104 (17.2)	93 (15.4)	124 (20.5)
Belief that vaccines are effective	24 (4.0)	44 (7.3)	74 (12.3)
Belief that diseases are dangerous for a baby already born	16 (2.7)	50 (8.3)	99 (16.4)
If the Kenya ministry of health recommended it	16 (2.7)	31 (5.1)	48 (8.0)
If a friend/relative/neighbor recommended it	5 (0.8)	12 (2.0)	22 (3.6)
If another pregnant woman recommended it	3 (0.5)	7 (1.2)	16 (2.7)
For ethical/moral reasons or recommended by local or religious leaders	1 (0.2)	1 (0.2)	4 (0.7)
If an NGO recommended it	0 (0.0)	1 (0.2)	5 (0.8)
Reasons why a woman would NOT receive a vaccine during pregnancy			
Would never refuse vaccination against tetanus during pregnancy	308 (51.0)	3 (0.5)	0 (0.0)
If she sees the vaccine causes side effects to a mother or a child	91 (15.1)	37 (6.1)	9 (1.5)
Belief that the vaccine is not effective	34 (5.6)	35 (5.8)	20 (3.3)
Concerns that the vaccine would weaken your immune system	30 (5.0)	15 (2.5)	15 (2.5)
Concerns that the vaccine could be dangerous for a baby in the womb?	18 (3.0)	21 (3.5)	23 (3.8)
For medical reasons (immunocompromised, HIV+)	10 (1.7)	4 (0.7)	5 (0.8)
For religious reasons	9 (1.5)	4 (0.7)	5 (0.8)
For ethical or moral reasons	4 (0.7)	2 (0.3)	6 (1.0)
Her husband/household member does not authorize her to get the vaccine	4 (0.7)	2 (0.3)	4 (0.7)
Influence from a political leader	2 (0.3)	12 (2.0)	3 (0.5)
Don’t want to answer	2 (0.3)	0 (0.0)	0 (0.0)
Belief that a disease like tetanus is not dangerous for the mother	1 (0.2)	4 (0.7)	4 (0.7)
For cultural beliefs	1 (0.2)	4 (0.7)	1 (0.2)
Belief that a disease like tetanus is not dangerous for a baby already born	1 (0.2)	3 (0.5)	1 (0.2)
Belief that it’s better to suffer from the natural disease than be vaccinated	0 (0.0)	0 (0.0)	2 (0.3)
No response	0 (0.0)	428 (70.9)	501 (83.0)
Other	89 (14.7)	30 (5.0)	5 (0.8)
Priority benefit when deciding to get vaccinated			
Baby in womb	355 (58.8)	203 (33.6)	46 (7.6)
Mother	206 (34.1)	253 (41.9)	145 (24.0)
Baby after it is born	43 (7.1)	148 (24.5)	413 (68.4)

**Figure 2.**
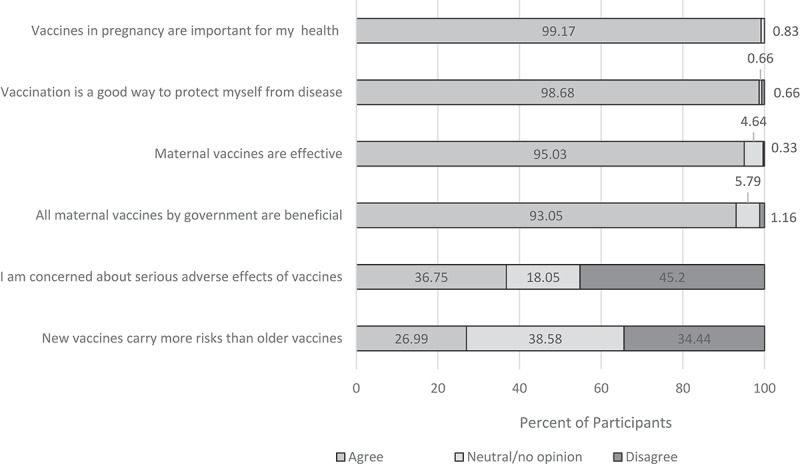
Pregnant Women’s Opinions on the Risks and Benefits of Maternal Vaccines

Nearly all the expectant mothers agreed with the statement that vaccines given in pregnancy are “important for my health” (n = 599, 99.2%) and that getting vaccinated is “a good way to protect myself from disease” (n = 596, 98.7%) (Figure 2). More than 90% of the mothers believed that maternal vaccines are effective and all vaccines offered by the government are beneficial. Overall, 36.8% expressed concern about serious adverse effects of vaccines and 27.0% agreed with the statement that new vaccines carry more risks than older ones.

**Figure 3.**
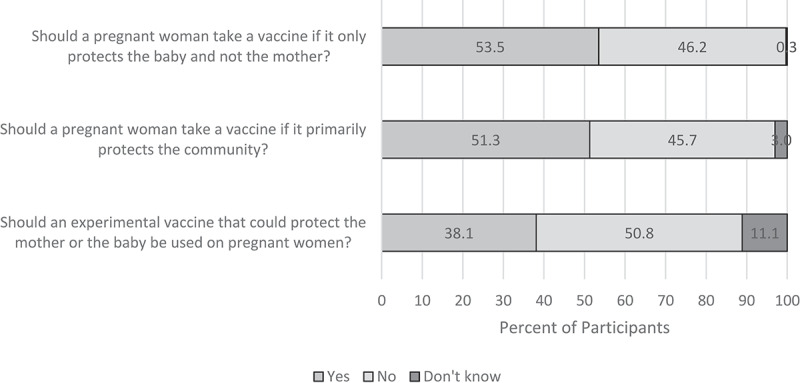
Pregnant Women’s Opinions on Vaccine Protection and Vaccine Priority During Pregnancy

Slightly more than a half (53.5%) of participants agreed that a pregnant woman should be vaccinated if only the baby (not the mother) is protected and 51.3% agreed that a pregnant woman should be vaccinated if it primarily protects the community; 38.1% agreed that an experimental vaccine that could protect the mother or baby should be used on pregnant mothers (Figure 3).

A small proportion of participants (n = 26, 4.3%) reported ever having refused a vaccine for either herself or a child in the past (Table 5). The most common reasons cited for refusal were not enough information (n = 11, 42.3%) and safety/side effects concerns (n = 9, 34.6%). Rumors about vaccine safety were mentioned by 3 (11.5%) mothers. A total of 26 (4.3%) mothers reported having a negative experience with a vaccine in the past, most of these (61.5%) were swelling at the injection site. Four (15.4%) women who reported a negative experience also reported ever refusing a vaccine in the past.

**Table 5.** Reasons for vaccine refusal, for either mother or baby, in the past and negative vaccine experiences among pregnant women enrolled in the study, N = 604

Characteristic	n	%
Ever refused a vaccine	26	4.3
Type of vaccine refused		
Tetanus	10	38.5
Polio	6	23.1
Measles	2	7.7
Cannot remember the name	4	15.4
Don’t know	3	11.5
Other	1	3.8
Reasons for refusal^a^		
Not enough information/not advertised	11	42.3
Didn’t think it was safe/side effects	9	34.6
Rumors about its safety	3	11.5
I did not think it was effective	3	11.5
Vaccine not important or necessary	3	11.5
Family/friends told me not to	2	7.7
Religious reasons	1	3.8
Distance to clinic	1	3.8
I think I was not at risk of the disease	1	3.8
There were no vaccines at the clinic	1	3.8
Other reasons	2	7.7
Ever had a negative experience (for mother or child) with a vaccine^a^	26	4.3
Swelling	16	61.5
Bad fever	6	23.1
Abscess	5	19.2

^a^Women could reply to more than one option so total >26.

When asked about influenza vaccine (not routinely available in Kenya), 402 (66.6%) agreed that a pregnant woman should be vaccinated, and 470 (77.8%) reported that if given the option, they would accept influenza vaccination (Table 6).

**Table 6.** Pregnant women’s knowledge on influenza, attitudes, and beliefs on influenza vaccination, N = 604

	Yes	Not sure	No
Knowledge, attitude/belief	n (%)	n (%)	n (%)
Have you ever heard about influenza?	453 (75.0)	5 (0.8)	146 (24.2)
Do you think that a pregnant woman should be vaccinated against influenza?	402 (66.6)	151 (25.0)	51 (8.4)
Is it likely for a pregnant woman who was not vaccinated against influenza to contract the disease?	373 (61.8)	159 (26.3)	72 (11.9)
Is a pregnant woman protected if she is vaccinated against influenza?	423 (70.0)	138 (22.9)	43 (7.1)
Do you think it is safe for a pregnant woman to receive the influenza vaccine?	373 (61.8)	203 (33.6)	28 (4.6)
Would a baby be protected against influenza if his/her mother received an influenza vaccine during pregnancy	334 (55.3)	191 (31.6)	79 (13.1)
If you were given the option to get an influenza vaccine, would you accept vaccination?	470 (77.8)	54 (8.9)	80 (13.3)

## Discussion

We found a high level of acceptance for maternal vaccines among pregnant women in Kenya. Most participants reported having received a vaccine during the current and/or prior pregnancy, and nearly all reported favorable views on the importance and effectiveness of vaccination during pregnancy. The main driver for vaccine acceptance in pregnancy was disease prevention, with protection of the baby in the womb as the highest priority. Few participants (<5%) reported having ever refused a vaccine for either themselves or a child, and the most commonly reported potential barrier to maternal vaccine acceptance was concern about side effects. Although influenza vaccine is not routinely administered to pregnant women in Kenya, nearly 80% of participants reported that they would accept it if offered. The findings demonstrate an important willingness on the part of pregnant women in Kenya to accept both currently available and potential future maternal vaccines.

The pregnant women enrolled in this study demonstrated an understanding of the role of vaccine in disease prevention. Most participants agreed that vaccines given in pregnancy are important for their health and that getting vaccines was a good way to protect themselves from disease. Knowledge about vaccines forms a critical component of vaccine literacy, which is the degree to which a person has the capacity to obtain, process, and understand basic vaccine information and services to help them make appropriate health decisions.^30^ For maternal vaccines, knowledge of vaccines among pregnant women has been shown to be an important driver of uptake in various settings.^16^ In this study, reported refusal of vaccines was very uncommon; however, among the small number (n = 26) that had refused a vaccine, lack of sufficient information was the most common reason for refusal provided. Thus, even in a setting where pregnant women are relatively knowledgeable about vaccines, it is important to ensure that adequate and appropriate information is shared about maternal vaccines. This might be particularly important for the introduction of maternal vaccines.

The primary factors found to facilitate vaccination during pregnancy were disease prevention, vaccine benefit to the baby in the womb, and recommendation by a health-care provider. An understanding of the role of vaccines in disease prevention is critical for vaccine acceptance. Studies in Nigeria^31^ and Ethiopia^32^ reported that women who had received TT during pregnancy most commonly cited protection against tetanus as the reason for vaccination. Recommendation by a health-care provider has very consistently been shown to impact pregnant women’s decision to be vaccinated^4,33-37^ although data from other African settings are limited. Nearly 60% of women in this study had received a recommendation for vaccination in their current pregnancy, and health-care providers were the most frequent source of vaccine recommendation. Of note, relatively few women reported having received a recommendation for vaccination from a community health worker, despite efforts by the Kenyan government to have community health workers provide outreach and communication on maternal and neonatal health services, including vaccination.^38,39^ Community health workers represent an underutilized resource for promoting maternal vaccination, particularly in areas with limited numbers of health-care providers.

The main potential barrier to maternal vaccination that emerged was concerns about vaccine safety. More than one-third of participants were concerned about serious adverse effects of vaccines. Side effects were also cited as the leading reason why a pregnant woman might refuse vaccination. Concerns about the safety of vaccines in pregnancy is a well-recognized barrier to the implementation of maternal vaccines globally.^13,40^ The safety of TT in Kenya was aggressively questioned in 2014 by the Kenya Conference of Catholic Bishops, who alleged that it was being used to sterilize women.^41^ Nonetheless, a high proportion of women in this study reported having received TT during a prior and/or current pregnancy. Thus, our findings suggest that the allegations did not substantially affect the acceptance of TT among pregnant women in Kenya. Nonetheless, in designing and implementing maternal vaccination programs, it is important to recognize that vaccine adverse effects are a primary concern among pregnant women.

New maternal vaccines are under development (e.g. against Group B *Streptococcus* and respiratory syncytial virus)^19-23^ and efforts are underway to increase the use of currently available but underutilized vaccines for pregnant women in LMIC (e.g. influenza and pertussis vaccines^17,18^). Most pregnant women in this study reported a willingness to receive influenza vaccine, which is not currently in routine use in Kenya, but was recommended by the Kenya National Immunization Technical Advisory Group in 2016, pending availability of local data on burden of influenza in pregnancy.^42^ Contrarily, 27.0% felt that new vaccines are riskier than old vaccines, and 50.8% reported that “experimental” vaccines should not be used on pregnant women. Also, 16.7% of participants reported they would only accept one vaccine during pregnancy, and 11.9% would accept up to two vaccines. Planning for introduction of new maternal vaccines should take into account perceptions of risk; combined antigen vaccines (i.e. fewer number of doses administer) may facilitate greater acceptance.

The study had several limitations. First, we used convenience sampling technique to reach study participants and had 16% of the women recruited through referrals which presents potential bias for similar knowledge, attitudes, and beliefs. Also, we recruited participants in only four counties and 48.8% of women enrolled were of Luo ethnicity. Nationally, the Kenyan population is made up of 42 tribes, and Luos represents 11% of the population.^43^ Although we aimed to enroll 100 women per site from health facilities, low numbers of patients in clinics at some sites (Mombasa and Marsabit), resulted in variability in numbers enrolled per site and per facility. Thus, our findings may not be generalizable to all of Kenya. Some questions involved health practices in past pregnancies and recall may have been inaccurate. Furthermore, participants may have given socially desirable responses, especially for questions regarding TT vaccination, which is recommended in Kenya. The data we captured on receipt of TT are not directly comparable to government statistics on the coverage of two or more TT doses among pregnant women in Kenya, and this study was not intended to assess vaccine coverage. However, it is notable that we observed high levels of vaccine acceptance when compared with the low TT coverage reported among pregnant women in Kenya;^9^ this contrast may be due to the limitations in the generalizability of our findings or the potential for socially desirable responses. Because the only maternal vaccine accessible to all pregnant women was TT, we could not reliably assess attitudes and behaviors regarding other maternal vaccines.

## Conclusion

This study adds to the body of data on factors which may facilitate or impede vaccine uptake among pregnant women in Africa and other LMICs, where such data remain sparse. Nationally, Kenya has made tremendous effort toward achieving the target coverage of 80% for TT vaccination in pregnancy. Opportunities for further improving uptake of maternal vaccines lie in providing adequate information about vaccines for pregnant women and mitigating safety concerns. Health-care providers are the main source of vaccine recommendation for pregnant women and must be encouraged to consistently advise antenatal patients about vaccination. Community health workers should be better leveraged to reach pregnant women with accurate messages about vaccination. Our findings suggest that new maternal vaccines introduced in Kenya, such as the influenza vaccine, will likely be well accepted by pregnant women, as long as information on benefits and risk is communicated widely and comprehensively.

## Supplementary Material

Supplemental Material
