# Supplementary material for: Drivers and barriers of vaccine acceptance among pregnant women in Kenya
Source: Hum Vaccin Immunother. 2020 Mar 25;16(10):2429–37. doi: 10.1080/21645515.2020.1723364 (PMC7644175; doi:10.1080/21645515.2020.1723364)
Supplement: Supplemental Material [file KHVI_A_1723364_SM1026.docx]

**Knowledge, Attitudes and Beliefs (KAB) Survey for Pregnant Women**

**SECTION A: Demographic information**

| 1 | What is your date of birth? | _ _ |
| --- | --- | --- |
| 2 | What is your level of education? | 1. No formal education and cannot read & write  2. No formal education but can write and read  3. Primary School  4. Secondary School  5. Polytechnics/other colleges  6. University  7. Vocational colleges |
| 3 | What is your marital status? | 1. Single  2. Married  3. Cohabitation  4. Divorced/ Separated  5. Widow  6. I don’t want to answer |
| 4 | What is your primary source of income? | 1. Subsistence farming  2. Commercial farming  3. Fishing  4. Housewife  5. Salaried worker (eg. teacher, nurse, office)  6. Small business (eg. sell maize) (Description-does not have a premise)  7. Business owner (eg. duka, kiosk) (Description-has a premise)  8. Skilled labor (eg. carpenter, tailor, jua kali)  9. Unskilled labor (eg. shamba, construction)  10.Student  11. Not Working(Probe)  12. Other: __________ |
| 5 | What is your religion? | 1. Catholic 2. Protestant 3. Traditional African Churches 4. Muslims 5. Hindu 6. Traditional Religion |
| 6 | What is your mother tongue? | 1. Luo  2. Kikuyu  3. Luhya  4. Kamba  5.Borana/Rendile/Burji/Somali  6.Swahilli  7. Mijikenda  8. Other: ________________ |

**SECTION- B**

| 7 | How many children currently live in your household? | | | | | |  | | | | |  |
| --- | --- | --- | --- | --- | --- | --- | --- | --- | --- | --- | --- | --- |
| 7a | How many are under five years? | | | | | |  | | | | |  |
| 8 | How many times have you been pregnant including this pregnancy? | | | | | | _ _ | | | | |  |
| 8a | Has any of your pregnancies resulted to miscarriage? | | | | | | 1. Yes 2. No. | | | | |  |
| 8b | What month of pregnancy are you in for this pregnancy? | | | | | |  | | | | |  |
| 8c | How many times have you visited the ANC? (include current visit if at clinic) | | | | | | -- | | | | |  |
| 8d | Have you been hospitalized during this pregnancy? | | | | | | 1. Yes  2. No | | | | |  |
| 8e | If yes, why? | | | | | | Write here | | | | |  |
| 9 | Has anyone recommended you to get vaccinated during your pregnancy? | | | | | | 1. Yes  2. No | | | | |  |
| If No or Not sure, skip to 10 | | | | | | | | | | | |  |
| 9a | Who recommended you to get vaccinated during this pregnancy? (Mark all that apply) | | | | | | 1. Doctor, Nurse, or other health care providers  2. Community Health Worker  3. Friend /Neighbor  4. Relative  5. Chemist  6. Through Radio, TV Or Internet/Social Media  7. Religious leaders  8. local leaders  9. Trained/Traditional Birth Attendants  10. Ministry of Health  11. Alternative Medicine Providers  12. Other:_________________________ | | | | |  |
| 9b | Which vaccine/s have you been recommended to get during this pregnancy? (mark all that apply) | | | | | | 1. Tetanus vaccine  2. Influenza vaccine  3. Pertussis vaccine  4. Human Papilloma Virus Vaccine  5. I do not remember the name  6. I don’t know  7. Other: ____________________________ | | | | |  |
| 10 | Did you receive a jab in your upper arm during this pregnancy (point to arm)? | | | | | | 1. Yes  2. No  3. Not sure | |  | | |  |
| If Yes, record details (preferably from ANC booklet. If not possible, get verbal report. List the source of information | | | | | | | | | | | |  |
|  | Vaccine | | | Shots | | | Approximate week of pregnancy | | Complications or problems | | |  |
|  | A | Tetanus | | |  | | |  | |  | | |
|  | B | Influenza | | |  | | |  | |  | | |
|  | C | Pertussis | | |  | | |  | |  | | |
|  | D | Human Papilloma Virus | | |  | | |  | |  | | |
|  | E | Don’t know | | |  | | |  | |  | | |
|  |  |  |  |  |  |  |  |  |  |  |  |  |
| 10a | In previous pregnancies, did you receive a jab in your upper arm (point to arm)? | | | | | 1. yes  2. no  3. not sure | | | | | |  |
| If no or not sure, skip to section C | | | | | | | | | | | |  |
| 10b | Which vaccine/s did you receive in any of your previous pregnancies?  (record all mentioned) | | | | | 1. Tetanus vaccine  2. Influenza vaccine  3. Pertussis vaccine  4. Human Papilloma Virus Vaccine  5. I do not remember the name  6. I don’t know  7. Other: ____________________________ | | | | | |  |
| 11 | Is there a maximum number of vaccines that you would be willing to receive during pregnancy? | | | | | 1. yes  2. no (go to question 12)  3. Not sure | | | | | |  |
| 11a | If yes, how many vaccines would you be willing to receive? | | | | | _ _ | | | | | |  |
| 12 | At what stage of pregnancy are you more likely to take a vaccine? | | | | | 1. All throughout pregnancy 2. During the first three months 3. During the first six months 4. Only during the last three months | | | | | |  |
| **SECTION C: Moral Foundation Questions** | | | | | | | | | | | |  |
| Some vaccines given in pregnancy only protect the mother, or only protect the baby, or both. | | | | | | | | | | | |  |
| 13 | Should a pregnant woman take a vaccine if it only protects the baby and not the mother? | | | | | | 1.yes  2.no  3. I do not know | |  | | |  |
| 13a | if it helps prevent spread of diease in the community but may not offer direct benefits for the woman or her child"? | | | | | | 1.yes  2.no | |  | | |  |
| 13b | Should an experimental vaccine that could protect the mother or the baby be used on pregnant women (after the safety of the vaccine has been tested)? | | | | | | 1.yes  2.no  3. I do not know | |  | | |  |
| 13c | When deciding to get a vaccine, whose benefit do you prioritize first, second and third between - the mother, the baby in the womb, and the baby after it is born (list in order of top priority) | | | | | | 1.write in  2.wriet in  3.write in | |  | | |  |
| 14 | Have you ever refused a vaccine (for you or your child)? | | | | | | 1. Yes  2. No  3. Not sure | |  | | |  |
| 14a | If yes, Which Vaccine? | | | | | | 1. Write in | |  | | |  |
| If no or not sure skip to Q14c | | | | | | | | | | | |  |
| 14b | What are the reasons why you refused a vaccine? (mark all that apply) | | | | | | 1. distance to clinic  2. there were no vaccines at the clinic (unavailable)  3. cost  4. didn’t think it was safe/side effects  5. religious reasons  6. cultural reason  7. family/friends told me not too  8. I did not think it was effective  9. Not enough information  10. Do not like injections  11. Not important or necessary  12. I think I was Not at risk of the disease  13. Other: ____ | | | | |  |
| 14c | What would make you not receive a vaccine? (Prompted choice; mark all that apply)  Probe to get more | | | | | | 1. You have concerns that the vaccine could weaken your immune system  2. You believe that it is better to suffer from the natural disease than to be vaccinated  3. You believe that the disease is not dangerous for yourself  4. You believe that the disease is not dangerous for a baby already born  5. You believe that the vaccine is not effective  6. For ethical or moral reasons  7. For religious reasons  8. For cultural beliefs  9.You have concerns that the vaccine could be dangerous for the baby who is in the womb  10. Your husband or household member does not authorize you to get the vaccine  11. For medical reasons (immunocompromised, HIV +)  12. I would never refuse vaccination against tetanus during pregnancy.  13. Influence from political leaders  14. If I see the vaccine causing side effects to a mother or child.  15. I don’t want to answer | | | | |  |
| 14d | Please give the top 3 reasons why you would NOT receive a vaccine during pregnancy. (mark all that apply; write in question) | | | | | | Drop-down created by the programmer based on choices in section 14c | | | | |  |
| 14e | Please give the top 3 reasons why you would receive a vaccine during pregnancy. (mark all that apply; write in question) | | | | | | 1. If a doctor, nurse or other health care providers recommended it. 2. If the Kenya Ministry of Health recommended it 3. If an NGO recommended it. 4. If a friend/relative/neighbor recommended it. 5. If another pregnant woman recommended it. 6. Vaccines help prevent diseases. 7. You believe that diseases are dangerous for a baby already born. 8. You believe that vaccines are effective. 9. For ethical/moral and or recommended by local or religious leaders. 10. The vaccines is beneficial to the baby in the womb. 11. The vaccines are offered for free/affordable, accessible and available. 12. I believe vaccines are important and safe | | | | |  |
| 15 | In your opinion, how much do you trust the following sources of information with regards to vaccination? | | | | | | | | | | |  |
| Source/Recommendation by | | | | | | | High | | Medium | | Low |  |
|  | A | | Doctor or nurse | | | |  | |  | |  |  |
|  | B | | Community Health Worker | | | |  | |  | |  |  |
|  | C | | Alternative medicine practitioner/traditional healers | | | |  | |  | |  |  |
|  | D | | Relative | | | |  | |  | |  |  |
|  | E | | Friend/Neighbor | | | |  | |  | |  |  |
|  | F | | Religious leader | | | |  | |  | |  |  |
|  | G | | Local leader | | | |  | |  | |  |  |
|  | H | | TV | | | |  | |  | |  |  |
|  | I | | Radio | | | |  | |  | |  |  |
|  | J | | Newspaper, Magazine | | | |  | |  | |  |  |
|  | K | | Internet/Social Media | | | |  | |  | |  |  |
|  | L | | Ministry of health | | | |  | |  | |  |  |
|  | M | | Trained/Traditional Birth Attendants | | | |  | |  | |  |  |
|  | N | | Chemist | | | |  | |  | |  |  |
|  | O | | Other: ______________ | | | |  | |  | |  |  |
| 16 | Who influences your decision to get a vaccine during pregnancy? (prompted question; record all mentioned) | | | | | | 1. Self  2. Husband  3. Your mother  4. Your father  5. Mother of the father of the baby (mother in law)  6.Father of the father of the baby (father in-law)  7. Other relatives  8. Friends  9. Another pregnant woman  10. Doctor, nurse, or other healthcare provider  11. the Kenyan Ministry of Health recommended it.  12. An NGO (e.g. World Health Organization) recommended it.  13 If an Alternative medicine practitioner (Need local term) recommended it  14. Religious Leader  15. Local leaders  14. Other: __________ | | | | |  |
| 16a | Out of the previous list, who are your top three influencers when deciding to get a vaccine during pregnancy? | | | | | | Combo box with a list of people who influence her decision based on choices in question 16. | | | | |  |
| Only ask 17 and 17a if they record they have children | | | | | | | | | | | |  |
| 17 | Who influences your decision to have a child vaccinated? | | | | | | 1. Self  2. Husband  3. Your mother  4. Your father  5. Mother of the father of the baby (mother in law)  6.Father of the father of the baby (father in-law)  7. Other relatives  7a. Co-wives  8. Friends  9. Another pregnant woman  10. Doctor, nurse, or other healthcare provider  11. The Kenyan Ministry of Health recommends it.  12. An NGO (e.g. World Health Organization) recommended it.  13 If an Alternative medicine practitioner (Need local term) recommended it  13. Local leaders  14. Religious Leaders  15. Other: | | | | |  |
| 17a | Out of the previous list, who are your top three influencers when deciding to have your child vaccinated? | | | | | | Combo box with choices from question 17 | | | | |  |

**SECTION D: Opinion questions**

| 18 | Do you think that it is difficult for some religious groups in your community/region to get vaccinations? | | | 1. yes  2. no  3. I don’t know |
| --- | --- | --- | --- | --- |
| 18a | If yes, record why | 1. Some religions associate vaccines with family planning and sterility.  2. Vaccines reduce the intelligence of a baby.  3. No belief in conventional medicine.  4. Belief in natural care in pregnancy and child care.  5. Protection is from God and they live by faith.  6. Vaccines can cause paralysis.  7. Vaccines can cause miscarriage.  8. Vaccines are against religious doctrines.  9. Ignorance about vaccines/immunization  10. Others _______________specify | | |
| 18b | Do you think that it is difficult for some ethnic groups in your community/region to receive vaccinations? | | | 1. yes  2. no  3. I don’t know |
| 18c | If yes, record why | 1. Tradition of no vaccination across generations.  2. Some ethnic groups have suffered from Adverse Events related to vaccines.  3. Misconceptions on vaccines.  4.Traditional practices and cultural beliefs  5. Others: Specify__________________ | | |
| 19 | Do religious leaders in your community support vaccines for pregnant women? | | | 1. Yes  2. No  3. Some  4. I don’t know |
| 19a | Do other leaders (i.e. political leaders, chiefs, health care workers) in your community support vaccines for pregnant women? | | | 1. yes  2. no  3. Some.  4. I don’t know. |
| 20 | Have you or your children ever had a negative experience with a vaccine? | | | 1. yes  2. no |
| 20a | If yes, What was the negative experience? | | | 1. Anaphylactic shock 2. Bad fever 3. Rash 4. Death 5. Abscess 6. Swelling 7. Convulsions 8. Others |
| 21 | Have you ever heard any rumors about the TT or flu vaccine? | | | 1.yes  2.no |
| If no, skip to 22 | | | | |
| 21a | If yes, record answers | |  | |
| 21b | Do you think these rumors are credible? | | | 1. Yes  2. No  3. I do not know |
| 21c | Did you still get vaccinated after you heard the rumors? | | | 1. Yes  2. No  3. Was not offered the vaccine |
| 22 | Do you think the TT vaccine causes infertility? | | | 1. Yes  2. No  3. I do not know |

23) For the following questions, please place an X in the appropriate box for each question, to indicate how much you agree with each statement (1 = ‘ Agree,’ 3 = Disagree’)

|  | 1  Agree | 2  Neutral/No opinion | 3  Disagree |
| --- | --- | --- | --- |
| A. Vaccines given in pregnancy are important for my health |  |  |  |
| B. all maternal vaccines offered by the government program in my community are beneficial |  |  |  |
| C. Maternal vaccines are effective |  |  |  |
| D. New vaccines carry more risks than older vaccines |  |  |  |
| E. Getting vaccines is a good way to protect myself from disease. |  |  |  |
| F. I am concerned about serious adverse effects of vaccines. |  |  |  |
| G. I do not need vaccines for diseases that are not common anymore. |  |  |  |

**Section E: Flu Questions**

| 24 | Have you ever heard about influenza? | 1. Yes  2. No  3. Not sure |
| --- | --- | --- |
| 25 | Do you think that a pregnant woman should be vaccinated against influenza? | 1. Yes  2. No  3. Not sure |
| 26 | If you were given the option to get a vaccine against influenza, would you accept vaccination? | 1. Yes  2. No  3. Not sure |
| 27 | Is it likely for a pregnant woman who was not been vaccinated against influenza to contract the disease? | 1. Yes  2. No  3. Not sure |
| 28 | Is a pregnant woman protected if she is vaccinated against influenza? | 1. Yes  2. No  3. Not sure |
| 29 | Do you think it is safe for a pregnant woman to receive influenza vaccine? | 1. Yes  2. No  3. Not sure |
| 30 | Would a baby after being born be protected against influenza, if his/her mother received an influenza vaccine during pregnancy? | 1. Yes  2. No  3. Not sure |
